# The positive effects of Ginsenoside Rg1 upon the hematopoietic microenvironment in a D-Galactose-induced aged rat model

**DOI:** 10.1186/s12906-015-0642-3

**Published:** 2015-04-15

**Authors:** Wenxu Hu, Pengwei Jing, Lu Wang, Yanyan Zhang, Jiadao Yong, Yaping Wang

**Affiliations:** Department of Histology and Embryology, Laboratory of Stem Cell and Tissue Engineering, Chongqing Medical University, No.1 Yixueyuan Road, Yuzhong District, 400016 Chongqing China; Department of stomatology and oral & maxillofacial surgery, YongChuan Hospital, Chongqing Medical University, 402160 Chongqing, China

**Keywords:** Hematopoietic, Bone marrow, Stem cell, BMSC, D-galactose, Aging, Senescence, Ginsenoside Rg1, Ginseng

## Abstract

**Background:**

Ginsenoside Rg1 (Rg1) is one of the most active ingredients in *Panax ginseng* and has been proven to have anti-oxidative and anti-aging properties. However, there have been few reports concerning the anti-aging effects of Rg1 on the hematopoietic microenvironment and bone marrow stromal cells (BMSCs).

**Methods:**

Thirty Sprague-Dawley rats were randomly divided into four groups (control, D-galactose (D-gal)-administration, Rg1-treatment, and D-gal-administration + Rg1-treatment groups). After D-gal and Rg1 treatment, BMSCs were extracted from femoral bone marrow for culture. After three passages, BMSCs were tested by senescence-associated β-galactosidase (SA-β-gal) staining, flow cytometric cell cycle phase distribution assay, CCK-8 cell proliferation assay, oxidative stress (reactive oxygen species [ROS], superoxide dismutase [SOD], and malondialdehyde [MDA]) assays, inflammatory marker (interleukin (IL)-2, IL-6, and tumor necrosis factor (TNF)-α) enzyme-linked immunosorbent assay (ELISA), stem cell factor (SCF) ELISA, and senescence-associated protein (p16, p21, and p53) Western blotting.

**Results:**

Compared to the D-gal-administration group, the D-gal-administration + Rg1-treatment group showed significantly decreased levels of SA-β-gal + cell %, ROS, MDA, inflammatory marker expression, and senescence-associated protein expression as well as significantly increased levels of S-phase %, cell proliferation, SOD activity, and SCF expression. Compared to controls, the Rg-1-treatment group displayed significantly reduced levels of SA-β-gal + cell %, G1 phase %, ROS, MDA, inflammatory marker expression, senescence-associated protein expression, and SCF expression as well as significantly increased levels of S-phase %, cell proliferation, and SOD activity.

**Conclusions:**

Rg1 improves the anti-aging ability of hematopoietic microenvironment through enhancing the anti-oxidant and anti-inflammatory capacities of BMSCs.

## Background

With the growing population and extended lifespan, aging has become a worldwide problem due to its substantial associated disability. For example, aging of the hematopoietic system and bone marrow probably leads to hematologic disease [1-3]. Bone marrow is the most important hematopoietic organ and is composed of hematopoietic cells and bone marrow stromal cells (BMSCs) at different stages of development. BMSCs, as the main component of the hematopoietic microenvironment, are where hematopoietic stem cells (HSCs) lodge, proliferate and differentiate. It is within the hematopoietic microenvironment that HSCs generate specific progenitor cells and the self-renewal divisions necessary to sustain themselves. The hematopoietic microenvironment may be sensitive to systemic senescence caused by natural aging or chemical induction. Thus, the senescence that occurs in the hematopoietic microenvironment and BMSCs may cause degenerative dysregulation of HSCs.

Traditional Chinese Medicine (TCM) theory indicates that the debility that occurs in bone marrow during natural aging is due to the degeneration of both “qi” and “blood”. Therefore, the principle for regulating bone marrow senescence should be based on invigorating both “qi” and “blood”. Ginsenoside Rg1 (Rg1) is one of the most active ingredients in *Panax ginseng* and has been proven to have various pharmacological actions in anti-oxidation and anti-aging [4]. Moreover, Rg1 has been extensively observed to enhance hematopoietic effects in mice and rats with drug and radiation-induced senescence, including the resitance to aging process of HSCs by inhibited the expression of p16 ^INK4a^ and p21 ^Cip1/Waf1^ both at gene and protein levels and the promotion to hematopoietic cytokines such as Stem Cell Factor (SCF) and Granulocyte-Macrophage Colony-Stimulating Factor (GM-CSF) [5,6].

The D-galactose (D-gal)-induced aged rat model is widely regarded as an ideal model to study mechanisms and screen drugs for HSC aging. Specifically, chronic systemic exposure of rodents to D-gal induces accelerated aging, including regression of bone marrow and the hematopoietic system that are similar to symptoms in natural aging [7,8]. In our previous study, we demonstrated that Rg1 possesses the capacity for anti-aging activity in HSCs both in vitro and vivo [9]. Although the aging of the hematopoietic microenvironment and BMSCs, as the supporting material of the HSCs, can also influence the hematopoietic function of bone marrow, there have been few reports concerning the anti-aging effects of Rg1 on the hematopoietic microenvironment and BMSCs. Thus, to better elucidate the underlying mechanism (s) of Rg1 in age-associated hematopoietic microenvironment degeneration, here we investigated the effects of Rg1 in the BMSCs of a D-gal-induced aged rat model.

## Methods

### Animals

Three month old male Sprague-Dawley rats were purchased from the Medical and Laboratory Animal Center of Chongqing (Chongqing, China) and housed in a temperature and light-controlled room with free access to water and food. All treatments were performed under sodium pentobarbital anesthesia, and all efforts were made to minimize animal suffering. The Committee on Ethics of Animal Experimentation at Chongqing Medical University (Chongqing, China) approved the protocols of this study prior to its implementation (Approval no.: 2013031).

Twenty rats were randomly divided into four groups (control group, D-gal-administration group, Rg1-treatment group, and D-gal-administration + Rg1-treatment group). In the D-gal-administration group, D-gal (120 mg/kg · d) was injected subcutaneously daily into rats for 42 days. In the D-gal-administration + Rg1-treatment group, ginsenoside Rg1 (20 mg/kg · d) was injected peritoneally daily concomitantly for 28 days from day 15 of D-gal injection. All control animals were given saline in the same volume subcutaneously and peritoneally, respectively. In the Rg1-treatment group, saline at the same volume with D-gal injection was injected subcutaneously for 42 days, and Rg1 (20 mg/kg · d) was injected peritoneally for 28 days from day 15 of saline injection.

### Reagents

Ginsenoside Rg1 (RSZD-121106, Purity = 98.3%) was purchased from Xi’an Haoxuan Biological Technology Co., Ltd (Xi’an, China), dissolved in ddH2O at the concentration of 20 mg/ml, and sterilized by ultrafiltration. The SOD kit and MDA kit were purchased from Nanjing Jiancheng Bioengineering Institute (Nanjing, China). The IL-2 kit, IL-6 kit, and goat anti-rabbit secondary antibody were purchased from Wuhan Boster Bio-engineering Co., Ltd. (Wuhan, China). The TNF-α Kit was obtained from Uscn Life Science Inc. (Wuhan, China). The BCA kit and SA-β-gal Staining kit were purchased from Beyotime Institute of Biotechnology (Shanghai, China). The anti-β-tubulin III antibody was obtained from Sigma Co. LLC. The anti-GFAP antibody was purchased from Wuhan Sanying Biotechnology Inc. (Wuhan, China).

### Sample collection

The bone marrow cells were collected according to Raghavendran’s method [5]. The femoral bones were separated out of the body, briefly soaked in 75% alcohol, and flushed three times in phosphate buffer solution (PBS) containing antibiotics (Penicillin–Streptomycin) under sterile conditions. The epiphyses of each bone were cut off. Bone marrow cell suspensions were prepared by flushing the diaphysis with PBS through syringe needles for three to five times. Then, full bone marrow cells were implanted in 25-cm^2^ tissue flasks containing 5 ml DMEM supplemented with 10% FBS and 1% penicillin-streptomycin. The cells were incubated in a 37°C, 5% CO_2_-humidified chamber. After 72 h of incubation, the supernatant and unattached cells were removed. The adherent cells were fed with fresh medium and incubated for an additional two days. The medium was pipetted off and discarded, the cells were trypsin-digested and detached, and then passaged to a new flask. The cultures were fed every three days by replacement of the medium. After three passages, BMSCs achieved 80% confluence. The confluent cell layers were washed twice with PBS. Adherent BMSCs were detached with 0.25% trypsin with EDTA and collected for further assays.

### Senescence-Associated β-Galactosidase (SA-β-gal) cytochemical staining

SA-β-gal is one of the most widely used biomarkers for aging cells [7,8]. The BMSCs were collected at passage three, and the SA-β-gal staining was performed according to the manufacturer’s instructions. Briefly, 5 × 10^4^ purified BMSCs from each animal were inoculated onto a six-well plate (with one slide per each well) and incubated for three days. Slides were washed twice with PBS, fixed in Fixative Solution for 10 min at room temperature, and stained with Staining Solution for 12 h at 37°C without CO_2_. Approximately 1 × 10^4^ cells were separated on each slide, and 400 cells were analyzed for each animal. The percentage of SA-β-gal-positive cells was calculated by counting the number of blue cells under bright field illumination and then calculating the ratio of SA-β-gal staining positive cells (%) in each group.

### Cell cycle phase distribution assay by flow cytometry

BMSCs were collected at a concentration of 5 × 10^5^ cells/ml and fixed with 70% cold ethanol at 4°C overnight. After centrifugation at 1000 rpm for 5 min, cells were incubated with propidium iodide (PI) at 4°C for 30 min in the dark and were subjected to flow cytometry using a FAC Scan Flow cytometer (BectonDickinson, New Jersey, USA) to determine the percentage of cells in each cell cycle phase.

### CCK-8 cell proliferation assay

For the quantitative determination of cellular proliferation and viability, we performed the CCK-8 assay. This assay was performed after BMSCs were collected at passage three. The cells were washed, counted, and seeded at a density of 4 × 10^5^ cells/ml per well in 96-well plates. At 24, 48, 72, 96, and 120 h after BMSC seeding, CCK-8 solution was added 4 h prior to the end of incubation. Cell viability was measured with a spectrophotometer at an absorbance of 450 nm.

### Inflammatory cytokine and Stem Cell Factor (SCF) detection in BMSCs by ELISA

The supernatant of the cell culture on passage three was collected, and levels of three inflammatory cytokines (IL-2, IL-6, and TNF-α) as well as SCF in each group were measured by ELISA according to the kit manufacturer’s instructions.

### Detection of oxidation-associated biomarkers

At passage three, BMSCs were collected and lysed in an ice bath for 30 min. After centrifugation (12000 rpm, 4°C, 30 min), the supernatant was collected. SOD activity -- determined using the commercially-available WST-1 assay kit -- and MDA content were detected by chemical colorimetric analysis according to the kit manufacturer’s instructions. The SOD activity assay is based on its ability to inhibit the oxidation of hydroxylamine by O_2_- produced by the xanthine-xanthineoxiase system. The results of the SOD activity assay were expressed as ratios of SOD suppression.

The thiobarbituric acid reaction (TBAR) method was used to determine the MDA, which can be measured at a wavelength of 532 nm by reacting TBA to form a stable chromophore. MDA content was expressed as nmol per milligram of BMSC protein. ROS production in BMSCs was measured using a DCFH-DA fluorescence flow cytometric assay. At passage three, the fluorogenic substrate solution was added to the medium and incubated at 37°C and 5% CO_2_ for 45 min. After being washed with cold PBS, the cells were quickly analyzed by FACS Calibur (BectonDickinson, USA).

### Western blotting analysis

BMSCs in each group were collected at passage three. Total protein was extracted, and the concentrations were measured by a BCA procedure. Samples containing 50 μg protein were separated on SDS-PAGE and transferred to PVDF membranes. Membranes were incubated overnight at 4°C with anti-p16, anti-21, and anti-53 antibodies (all diluted 1:500). The secondary antibody was diluted 1:5000 in TBST. The membranes were visualized using the enhanced chemiluminescence (ECL) detection system (Pierce, USA). β-actin was used as an internal control. Integral optical density (IOD) was quantified using Image Pro Plus (Media Cybernetics).

### Statistical analysis

SPSS v17.0 was used for all statistical analyses. One-way ANOVA was used for comparing mean values across groups, and multiple comparisons were made using the LSD test. Differences were considered significant at *P* < 0.05.

## Results

### Rg1 reduces SA-β-Gal staining of BMSCs in aged rats

**Figure 1:**
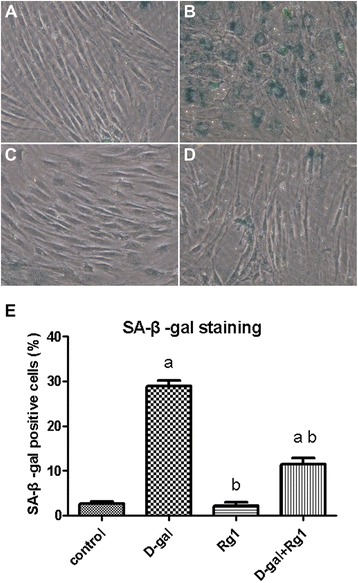
Rg1’s Effect upon Senescence-Associated β-Galactosidase Staining. The BMSCs were collected on day 5 of passage 3. The control and Rg1-treatment group showed normal structures, while D-gal-administration group showed that aged cells are stained in blue in the cytoplasm. Rg1 treated group exhibited a small number of blue stained cells. All the figures of BMSCs were shown with the light microscope at 200 times magnification. **(A)** The control group, **(B)** D-gal-administration group, **(C)** Rg1-treatment group, and **(D)** D-gal-administration + Rg1-treatment group. **(E)** a: comparison with the control group (*p* < 0.05); b: comparison with the D-gal-administration group (*p* < 0.05). Each error bar represents the standard deviation (SD).

The intensity of SA-β-gal staining was evaluated by means of the percentage of SA-β-gal positive cells (%) (Figure 1). The percentages of SA-β-gal positive cells were not significantly different between the control group and the Rg1-treatment group (Figure 1). As expected, the D-gal-administration group induced a significant increase in the percentage of SA-β-gal positive cells compared to that of the control group (*p* < 0.05; Figure 1). However, in the D-gal-administration + Rg1-treatment group, the percentage of SA-β-gal positive cells was significantly reduced (*p* < 0.05; Figure 1). These findings suggest that Rg1 can protect BMSCs against senescence.

### Rg1 affects cell cycle phase distribution of BMSCs in aged rats

**Figure 2:**
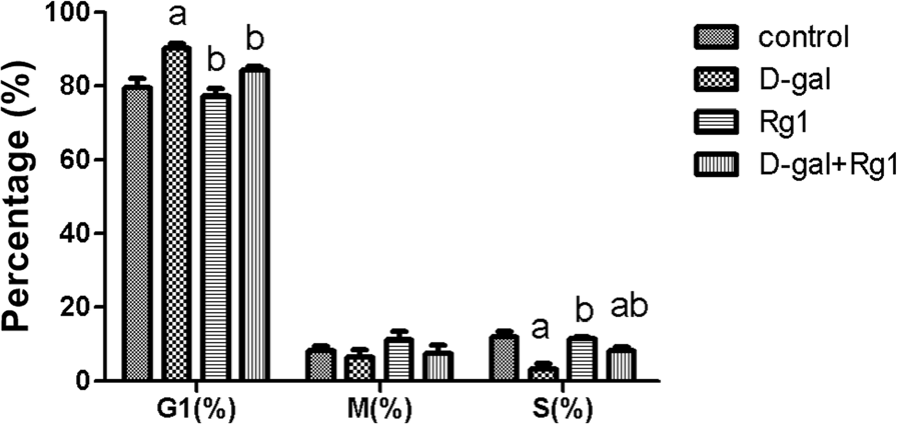
Rg1’s Effect upon the Cell Cycle Phase Distribution of BMSCs in Aged Rats. The BMSCs were collected on day 5 of passage 3. Cell cycle phase distribution was measured by flow cytometry with ≥5 × 10^5^ cells tested for each sample. a: comparison with the control group (*p* < 0.05); b: comparison with the D-gal-administration group (*p* < 0.05).

When compared to the D-gal-administration group, the BMSCs in both the Rg1-treatment and D-gal-administration + Rg1-treatment groups displayed G1-phase shortening, which is the reverse of the effect observed in the control group (*p* < 0.05; Figure 2). BMSCs from the D-gal-administration group displayed G1-phase arrest, and the percentages of cells in S phase were significantly decreased compared to that of the control group (*p* < 0.05; Figure 2). However, the percentages of cells in S phase in both the Rg1-treatment and D-gal-administration + Rg1-treatment groups were significantly elevated (*p* < 0.05; Figure 2). There were no significant differences among the four groups regarding cell percentages in M phase (Figure 2).

### Rg1 Promotes BMSC proliferation

**Figure 3:**
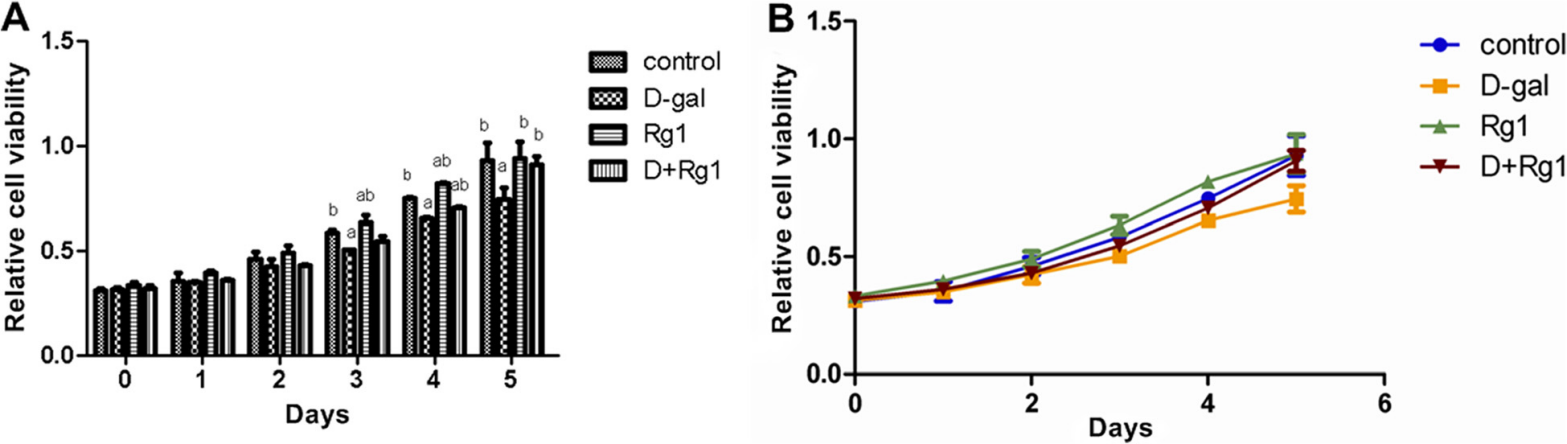
Rg1’s Effect upon Cell Proliferation of BMSCs in Aged Rats. The BMSCs were collected on day 5 of passage 3. **(A, B)** There were no differences among the four groups at days one and two by analysis of variance (ANOVA). However, at days three, four, and five, there were significant differences between the four groups by ANOVA. a: comparison with the control group (*p* < 0.05); b: comparison with the D-gal-administration group (*p* < 0.05). Each error bar represents the standard deviation (SD).

As expected, D-gal administration led to significant inhibition of BMSC proliferation compared to controls (*p* < 0.05; Figure 3). However, after Rg1 treatment, BMSC proliferation in the D-gal-administration + Rg1-treatment group was elevated significantly compared to the D-gal-administration group (*p* < 0.05; Figure 3).

### Rg1 affects inflammatory cytokine and SCF levels of BMSCs in aged rats

**Figure 4:**
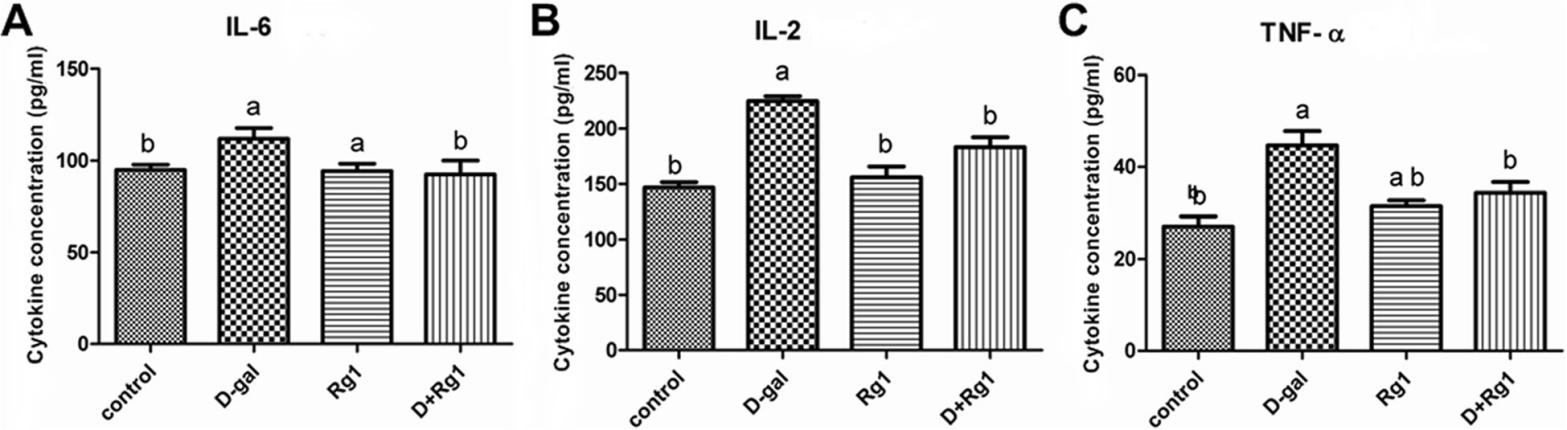
Rg1’s Effect upon Inflammatory Cytokine Secretion by BMSCs in Aged Rats. The BMSCs were collected on day 5 of passage 3. **(A)** Interleukin (IL)-6, **(B)** IL-2, and **(C)** tumor necrosis factor (TNF)-α. a: comparison with the control group (*p* < 0.05); b: comparison with the D-gal-administration group (*p* < 0.05). Each error bar represents the standard deviation (SD).

The levels of IL-2, IL-6 and TNF-α significantly increased, and SCF significantly decreased, in the BMSCs from the D-gal-administration group when compared with the control group (*p* < 0.05; Figure 4). However, the levels of the three inflammatory cytokines were significantly reduced in the D-gal-administration + Rg1-treatment group relative to the D-gal-administration group (*p* < 0.05; Figure 4). Conversely, the level of SCF was significantly elevated in the D-gal-administration + Rg1-treatment group compared to the D-gal-administration group. Simultaneously, Rg1 treatment did not significantly reduce the levels of IL-2, IL-6 and TNF-α in the Rg1-treatment group compared with controls, but the level of SCF was significantly promoted in the Rg1-treatment group compared with controls (*p* < 0.05; Figure 4). These findings indicate that inflammation in D-gal-induced aged BMSCs is alleviated, while SCF expression is promoted, by Rg1 treatment.

### Rg1’s anti-oxidative effects upon BMSCs in aged rats

**Figure 5:**
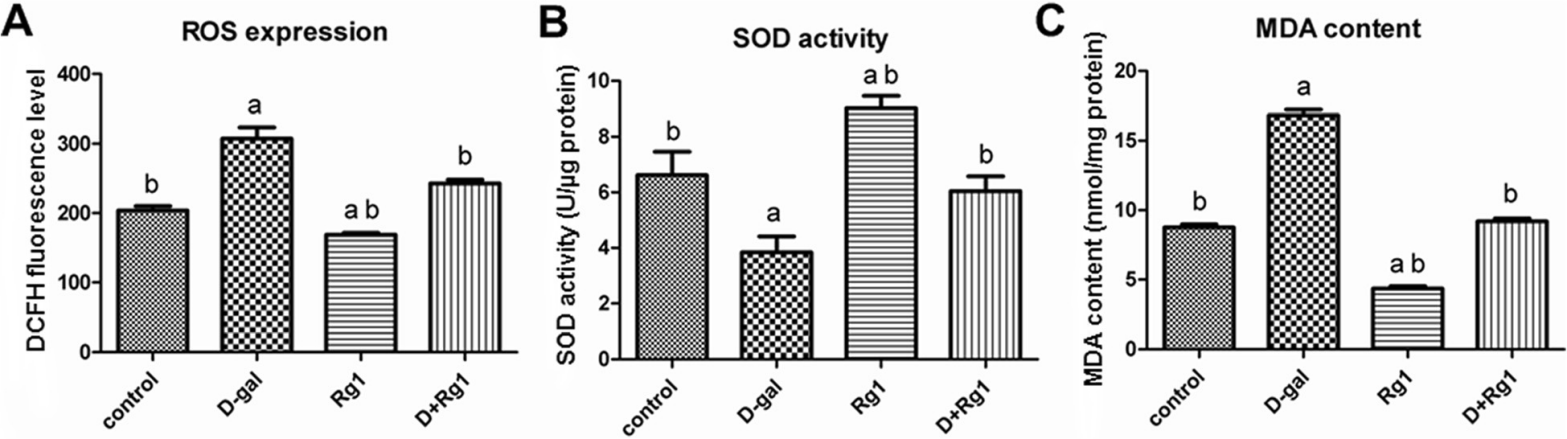
Rg1’s Effect upon Oxidative Stress and Anti-Oxidant Activity of BMSCs in Aged Rats. The BMSCs were collected on day 5 of passage 3. **(A)** Reactive oxygen species (ROS) levels, **(B)** superoxide dismutase (SOD) activity, and **(C)** malondialdehyde (MDA) content. a: comparison with the control group (*p* < 0.05); b: comparison with the D-gal-administration group (*p* < 0.05). Each error bar represents the standard deviation (SD).

The oxidative stress produced by reactive oxygen species (ROS) is one of the main causes of cell senescence. Superoxide dismutase (SOD) participates in the removal of ROS from the cellular environment, and malondialdehyde (MDA) is an end-product of ROS-induced peroxidation that is widely used as a marker of oxidative stress. We evaluated cellular ROS levels, SOD activity, and MDA content in the BMSCs to determine whether the anti-aging effects of Rg1 are mediated by alleviating oxidative stress. In the DCFH-DA assay, the cellular ROS levels were elevated significantly in the D-gal-administration group when compared with controls with significant inhibitory effects of Rg1 observed in the D-gal-administration + Rg1-treatment group (*p* < 0.05; Figure 5). In the Rg1-treatment group, the level of ROS production was not inhibited significantly compared to controls (Figure 5). When compared to the control group, the ratio of SOD suppression decreased significantly and the MDA content increased significantly in BMSCs in the D-gal-administration group (*p* < 0.05; Figure 5). Meanwhile, Rg1 significantly rescued the reduction in SOD activity, and partially rescued the increase in MDA content, in the D-gal-administration + Rg1-treatment group (*p* < 0.05; Figure 5). Interestingly, the Rg1-treatment group also significantly increased SOD activity and significantly reduced MDA content (*p* < 0.05; Figure 5), showing Rg1 exerts anti-oxidant effects by enhancing the activity of endogenous anti-oxidative defense enzymes.

### Rg1 affects p16, p21, and p53 expression in BMSCs of aged rats

**Figure 6:**
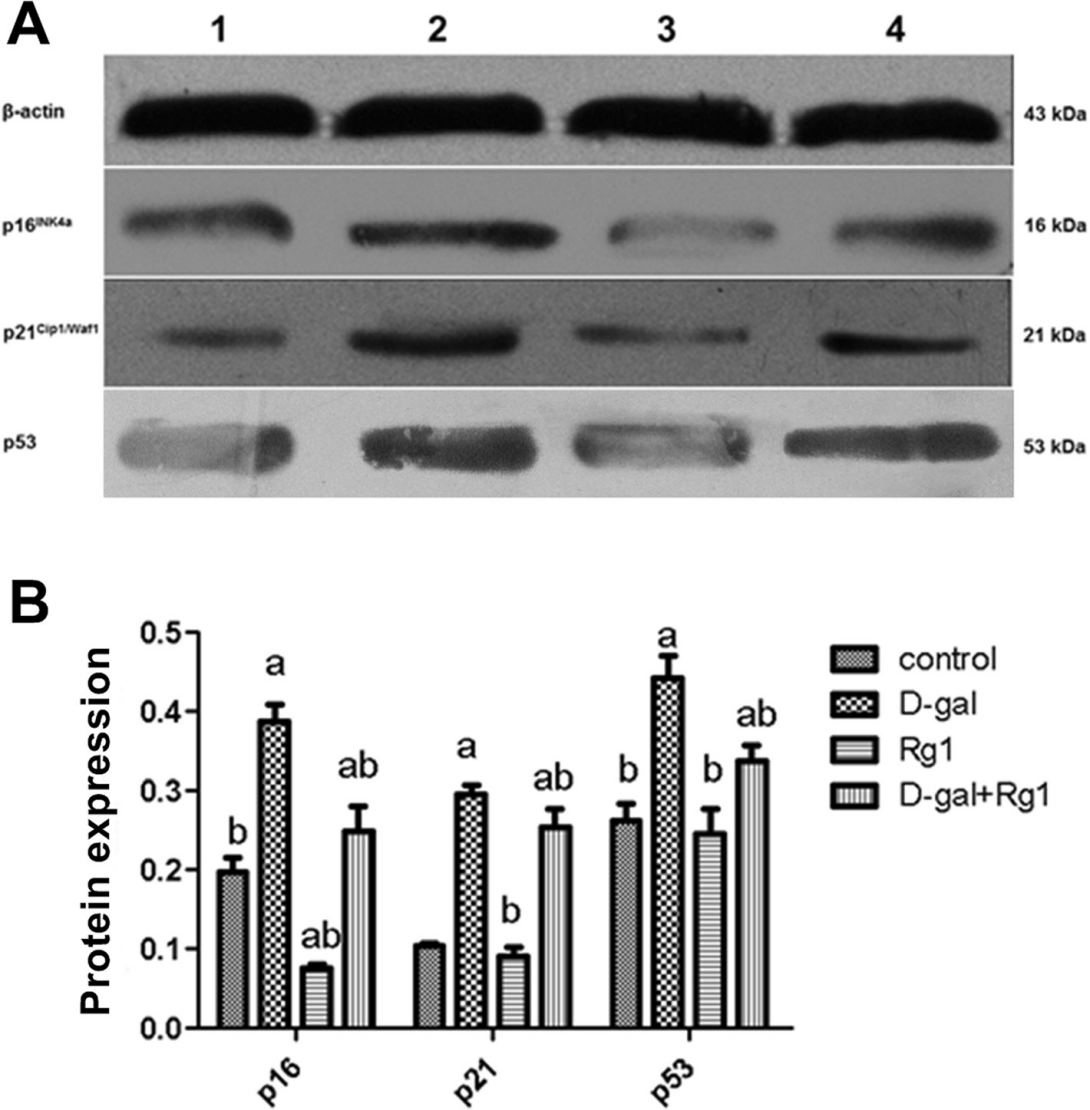
Rg1’s Effect on Senescence-Associated Protein Expression of BMSCs in Aged Rats. The BMSCs were collected on day 5 of passage 3. **(A)** Western blots showing β-actin, p16, p21, and p53 protein expression. **(B)** The integral optical density (IOD) values for p16, p21, and p53 proteins normalized to β-actin were calculated. a: comparison with the control group (*p* < 0.05); b: comparison with the D-gal-administration group (*p* < 0.05). Each error bar represents the standard deviation (SD).

The p16^INK4a^-retinoblastoma (Rb) pathway and the p19^Arf-Mdm2^-p53-p21^Cip1/Waf1^ pathway are two important signal transduction pathways involved in cell aging and cell cycle arrest. According to Western blotting analysis, the levels of p16^INK4a^, p21^Cip1/Waf1^, and p53 were significantly higher in the D-gal-administration group than in the control group (*p* < 0.05; Figure 6). With Rg1 treatment, the levels of these three proteins declined significantly (*p* < 0.05; Figure 6).

## Discussion

During natural aging, the hematopoietic system undergoes progressive morphologic and functional changes that occur in both the hematopoietic stem/progenitor cells and the hematopoietic niche, such as declines in hematopoietic capacity and tumorigenesis monitoring. Thus, it will be of great value to identify drugs that counteract hematopoietic degeneration in order to prevent certain blood diseases associated with aging. Our previous study demonstrated that Rg1 administration in mice enhances the resistance of HSC/HPCs to ionizing radiation-induced senescence by inhibiting oxidative stress reactions. However, the effects of Rg1 upon hematopoietic microenvironment aging remained unknown. Thus, to better elucidate the underlying mechanism (s) of Rg1 in age-associated hematopoietic microenvironment degeneration, here we investigated the effects of Rg1 on the BMSCs of a D-gal-induced aged rat model.

The cell cycle phase distribution of BMSCs reflects the proliferative activity of BMSCs and is an important indicator of hematopoietic-sustaining function. The overwhelming majority of cells within the bone marrow in a natural or drug-induced aging scenario remain in the G1 phase and cannot pass the G1/S check point [6], resulting in a decrease in the proliferation index. Accordingly, the decrease of BMSCs in aged rats could be due to an inhibition of a cell proliferation and/or an increase in cell apoptosis. To obtain a deeper insight into the mechanism (s) underlying the anti-aging activity of Rg1 in a D-gal induced aged rat model, we performed cell cycle phase distribution and apoptosis analysis of BMSCs through flow cytometry. As a result, we found significant decreases in BMSCs in the aged rat resulting from an inhibition of the cell proliferation as well as an increase in cell death following D-gal administration, which is in agreement with previous in vitro research on HSCs [5,7]. Moreover, we found that Rg1 facilitated BMSC proliferation, prevented BMSCs from proceeding to apoptosis, and also promoted the recovery of BMSCs. However, the detailed molecular mechanism (s) underlying Rg1’s effects upon cell cycle-related and apoptosis-related cellular pathways in the BMSC remains to be elucidated.

SCF is an acid glycoprotein secreted from stromal cells into the hematopoietic microenvironment and plays an important role in the hematopoiesis during embryonic development. All sites where hematopoiesis occurs, such as the bone marrow, express SCF. Mice die from severe anemia under conditions lacking SCF or SCF receptors [10]. SCF provides signalling cues that direct HSCs to their hematopoietic microenvironment and also plays an important role in HSC maintenance [10,11]. The present study indicates that Rg1 is able to elevate BMSC-secreted SCF in D-gal-induced aged rats, suggesting that Rg1 promotes hematopoietic function in aged rats by contributing to the maintenance of HSCs.

The foregoing results display that the positive effects resulting from the Rg1 treatment include promotion of cell proliferation, migration, and anti-apoptotic activity. We also evaluated anti-senescence in the D-gal-induced aged rat model by the aging biomarker SA-β-gal, which reflects the function of the lysosomes and accumulates in aging cells as lysosomes begin to malfunction. We found that Rg1 administration significantly decreased SA-β-gal expression in the bone marrow of aged rats, indicating that Rg1 is able to protect against BMSC senescence, which coincides with our previous in vitro study on Rg1’s effect on HSC senescence [6].

Aging has been associated with chronic inflammation [12-14]. When chronic inflammation occurs in aged bone marrow, a variety of BMSC products and inflammatory cytokines, such as IL-2, IL-6, and TNF-α, are released [15-17]. In the present study, Rg1 treatment significantly reduced the levels of IL-2, IL-6, and TNF- α in aged rats, suggesting that Rg1 can protect BMSCs from age-associated chronic inflammation.

Oxidative damage caused by ROS is one of key sources of cellular senescence [18-20]. ROS are formed as a natural by-product of the normal metabolism of oxygen and have important roles in cell signaling and homeostasis [21]. Additionally, ROS constitute one of the stimuli that induce cellular senescence [22]. Senescence occurs when there is an imbalance between increased ROS levels and decreased anti-oxidant defenses. When ROS production increases and/or ROS elimination downregulates, there will be an increase in ROS that results in lipidic oxidative damage to cellular function and structure [23]. MDA content reflects this lipidic oxidative damage in vivo, and SOD activity reflects antioxidant defense. In the present study, Rg1 promoted SOD activity while decreasing ROS and MDA content in both aged and control rats, suggesting that Rg1 induces anti-oxidant defense in the hematopoietic microenvironment of aged rats.

A variety of intracellular and extracellular stresses can promote cellular senescence [24]. These stressors – including oxidative stress, oncogene activation, and radiation stress -- engage various intracellular signaling pathways such as the p16^INK4a^-retinoblastoma (Rb) pathway and the p19^Arf-Mdm2^-p53-p21^Cip1/Waf1^ pathway. According to this study, ROS and MDA levels, as well as the senescence-related proteins (p16, p21, and p53) in aged rats were significantly higher compared to controls. Based on these combined findings, we speculate that the underlying mechanism of BMSC aging in the D-gal rat model is oxidative stress. Interestingly, Rg1 inhibited expression of these senescence-related proteins not only in aged rats but also in controls. Furthermore, activated p53 activates p21, which induces cell-cycle arrest by inhibiting cyclin E-Cdk2. Additionally, p16^INK4a^ inhibits cell-cycle progression by targeting cyclin D–Cdk4 and cyclin D–Cdk6 complexes. Both p21 and p16^INK4a^ preventing the phosphorylation of pRb, thus resulting in continued repression of E2F target genes required for S-phase onset. Thus, these senescence-related protein findings correspond to the findings from our cell cycle and cell proliferation assays.

## Conclusion

Rg1 improves the anti-aging ability of hematopoietic microenvironment through enhancing the anti-oxidant and anti-inflammatory capacities of BMSCs. Further research is needed to investigate the detailed molecular mechanism (s) underlying Rg1’s effects upon cell cycle-related and apoptosis-related cellular pathways in BMSCs.

## References

[1] Civini S, Jin P, Ren J, Sabatino M, Castiello L, Jin J (2013). Leukemia cells induce changes in human bone marrow stromal cells. J Transl Med.

[2] Ehteram H, Bavarsad MS, Mokhtari M, Saki N, Soleimani M, Parizadeh SM (2014). Prooxidant-antioxidant balance and hs-CRP in patients with beta-thalassemia major. Clin Lab.

[3] Drašar ER, Jiang J, Gardner K, Howard J, Vulliamy T, Vasavda N (2014). Leucocyte telomere length in patients with sickle cell disease. Br J Haematol.

[4] Zhu J, Mu X, Zeng J, Xu C, Liu J, Zhang M (2014). Ginsenoside Rg1 prevents cognitive impairment and hippocampus senescence in a rat model of D-galactose-induced aging. PLoS One.

[5] Raghavendran HR, Sathyanath R, Shin J, Kim HK, Han JM, Cho J (2012). Panax ginseng modulates cytokines in bone marrow toxicity and myelopoiesis: ginsenoside Rg1 partially supports myelopoiesis. PLoS One.

[6] Zhou Y, Wang JW, Jiang R, Yao X, Yang B, Cai SZ (2013). [Study on anti-aging effect of ginsenoside Rg1 in serial transplantation of hematopoietic stem cells and progenitor cells]. Zhongguo Zhong Yao Za Zhi.

[7] Dimri GP, Lee X, Basile G, Acosta M, Scott G, Roskelley C (1995). A biomarker that identifies senescent human cells in culture and in aging skin in vivo. Proc Natl Acad Sci U S A.

[8] Song X, Bao M, Li D, Li YM (1999). Advanced glycation in D-galactose induced mouse aging model. Mech Ageing Dev.

[9] Chen C, Mu XY, Zhou Y, Shun K, Geng S, Liu J (2014). Ginsenoside Rg1 enhances the resistance of hematopoietic stem/progenitor cells to radiation-induced aging in mice. Acta Pharmacol Sin.

[10] Broudy VC (1997). Stem cell factor and hematopoiesis. Blood.

[11] Bruunsgaard H, Andersen-Ranberg K, Jeune B, Pedersen AN, Skinhoj P, Pedersen BK (1999). A high plasma concentration of TNF-alpha is associated with dementia in centenarians. J Gerontol A Biol Sci Med Sci.

[12] Peng B, Wang C, Feng L, Wang YP: The effects and the underlying mechanisms of Ginsenoside Rg1 to regulate neural stem cell senescence. Chin J Cell Biol, 33:1116-1122

[13] Franceschi C, Campisi J (2014). Chronic inflammation (inflammaging) and its potential contribution to age-associated diseases. J Gerontol A Biol Sci Med Sci.

[14] Xu SF, Yu LM, Fan ZH, Wu Q, Yuan Y, Wei Y (2012). Improvement of ginsenoside Rg1 on hematopoietic function in cyclophosphamide-induced myelosuppression mice. Eur J Pharmacol.

[15] Ershler WB, Keller ET (2000). Age-associated increased interleukin-6 gene expression, late-life diseases, and frailty. Annu Rev Med.

[16] Mohebali D, Kaplan D, Carlisle M, Supiano MA, Rondina MT (2014). Alterations in platelet function during aging: clinical correlations with thromboinflammatory disease in older adults. J Am Geriatr Soc.

[17] Parmeggiani F, Sorrentino FS, Romano MR, Costagliola C, Semeraro F, Incorvaia C (2013). Mechanism of inflammation in age-related macular degeneration: an up-to-date on genetic landmarks. Mediators Inflamm.

[18] Fujihara M, Azuma H, Ikeda H, Yamaguchi M, Hamada H (2011). Bone marrow stromal cell line promotes the proliferation of mast cell progenitors derived from cord blood CD34+ cells under serum-free conditions with a combination of both cell-cell interaction and soluble factors. Artif Cells Blood Substit Immobil Biotechnol.

[19] Blouin R, Bernstein A (1993). The White Spotting and Steel Hereditary Anaemias of the Mouse. Freedman MH, Feig SA. Clinical Disorders and Experimental Models of Erythropoietic Failure.

[20] Rai P, Onder TT, Young JJ, McFaline JL, Pang B, Dedon PC (2009). Continuous elimination of oxidized nucleotides is necessary to prevent rapid onset of cellular senescence. Proc Natl Acad Sci U S A.

[21] Hodzic M, Naaldijk Y, Stolzing A (2013). Regulating aging in adult stem cells with microRNA. Z Gerontol Geriatr.

[22] Bentov Y, Casper RF (2013). The aging oocyte–can mitochondrial function be improved?. Fertil Steril.

[23] Devasagayam TP, Tilak JC, Boloor KK, Sane KS, Ghaskadbi SS, Lele RD (2004). Free radicals and antioxidants in human health: current status and future prospects. J Assoc Physicians India.

[24] van Deursen JM (2014). The role of senescent cells in ageing. Nature.

